# Vitamin D status and its relation to exercise performance and iron status in young ice hockey players

**DOI:** 10.1371/journal.pone.0195284

**Published:** 2018-04-09

**Authors:** Joanna Orysiak, Joanna Mazur-Rozycka, John Fitzgerald, Michal Starczewski, Jadwiga Malczewska-Lenczowska, Krzysztof Busko

**Affiliations:** 1 Department of Nutrition Physiology and Dietetics, Institute of Sport – National Research Institute, Warsaw, Poland; 2 Central Institute For Labour Protection – National Research Institute (CIOP- PIB), Department of Ergonomics, Warsaw, Poland; 3 Department of Kinesiology and Public Health Education, University of North Dakota, Grand Forks, North Dakota, United States of America; 4 Department of Physiology, Institute of Sport – National Research Institute, Warsaw, Poland; 5 Department of Anatomy and Biomechanics, Kazimierz Wielki University, Bydgoszcz, Poland; Medical University of Gdańsk, POLAND

## Abstract

**Objectives:**

The aim was to examine the association between serum vitamin D concentration and isometric strength of various muscle groups, vertical jump performance, and repeated sprint ability in young ice hockey players. The secondary aim was to determine the association between vitamin D deficiency and indices of iron status.

**Methods:**

Fifty male ice hockey players (17.2±0.9 years) participated in this cross-sectional study. Exercise performance was evaluated using isometric strength measures of upper and lower extremities, vertical jump performance and repeated sprint ability (RSA). Blood samples were collected for the determination of serum 25-hydroxyvitamin D (25(OH)D) and multiple indicies of iron status.

**Results:**

The mean serum 25(OH)D concentration was 30.4 ng·ml^-1^ and ranged from 12.5 to 91.4 ng·ml^-1^. Eleven participants (22%) had vitamin D deficiency and 20 athletes (40%) had vitamin D insufficiency. Serum 25(OH)D concentration was not positively correlated with isometric muscle strength, vertical jump performance, or RSA after adjusting for age, training experience, fat mass, fat free mass and height. Serum 25(OH)D concentration was not associated with indices of iron status.

**Conclusion:**

Vitamin D insufficiency is highly prevalent in ice hockey players, but 25(OH)D concentration but it is not associated with exercise performance or indices of iron status.

## Introduction

Vitamin D deficiency in athletes is common, especially among disciplines training indoors [1]. The presence of the vitamin D receptor in skeletal muscle, cardiac muscle and vascular tissue support the assertion that deficient vitamin D status may reduce physical performance in sport [2]. In athletes, serum 25-hydroxyvitamin D (25(OH)D) has been associated with strength, power [3], sprinting performance [4,5] and aerobic capacity [5]. However, not all investigations in athletes corroborate these findings [6,7,8] or find consistent relationships between 25(OH)D concentration and exercise performance [9,10].

The discrepancies in the literature may, in part, be due to selective use of exercise performance measures and differences in exercise training. It has been suggested that poor vitamin D status may impact the functioning of muscle groups differently due to fibre type predisposition [5] and that this effect may be modified by exercise training [9,10]. To date, investigations in athletes tend to employ only a few select measures of exercise performance and recruit cohorts of athletes with differing training histories and training practices at the time of study. Studies incorporating a comprehensive assessment of muscle function across muscle groups in a cohort of athletes with similar exercise training history and exposure are needed to evaluate this hypothesis. Furthermore, the association between 25(OH)D concentration and repeated sprint ability (RSA) has yet to be evaluated.

Vitamin D deficiency may also impact iron status and lead to anemia [11,12]. Iron deficiency is common among athletes [13,14] and many studies demonstrate that latent deficiency of this element may impair physical performance [14,15]. However, few studies have examined the relationship between 25(OH)D concentration and iron status in athletes [16,17].

The main purpose of this study was to assess the association between serum 25(OH)D concentration and isometric strength of the upper and lower extremities, vertical jump performance, and RSA in young ice hockey players from the same school. The secondary aim was to determine the association between 25(OH)D concentration and indicies of iron status. We hypothesized that a lower concentration of 25(OH)D would be associated with reduced exercise performance and impaired iron status.

## Methods

### Ethics statement

This study was approved by the local Ethical Committee at the Institute of Sport—National Research Institute in Warsaw, Poland (KEBN-16-18-JO) and conducted according to the Declaration of Helsinki. All participants (or their legal guardians if the athlete was under 18 years of age) gave their written consent to participate in the study and were informed about the purpose and test procedures. Furthermore they were aware of possibility to withdrawal of consent at any time for any reason.

### Participants

Fifty male ice hockey players participated in our cross-sectional study. The athletes were students from the Private Athletic High School of the Polish Ice Hockey Federation. The majority of athletes competed on the Polish national U18 and U20 teams and were recruited during competitive season in October from the Sosnowiec area, Poland (50°N). All athletes were medically examined and healthy. Descriptive physical characteristics of athletes is presented in Table 1.

**Table 1:** Descriptive characteristics for all ice hockey players (n = 50).

	Mean (SD)	Min-Max
Age (years)	17.2 (0.9)	15.6–18.7
Height (cm)	181 (7)	166–194
Body mass (kg)	75.6 (10.8)	52.4–100.8
BMI (kg·m^-2^)	23.1 (2.7)	17.2–28.4
Percent body fat (%)	12.9 (3.8)	3.5–20.8
Fat free mass (kg)	65.6 (7.5)	47.1–82.9
Fat Mass (kg)	10.0 (4.1)	2.0–19.4
25(OH)D (ng·ml^-1^)	30.3 (14.9)	12.5–91.4
Ferritin (ng·ml^-1^)	41 (22)	10–136
TIBC (μg·100ml^-1^)	336 (41)	243–507
sTfR (μg·ml^-1^)	5.5 (1.7)	3.7–15.2
Iron (μg·dl^-1^)	84.3 (28.5)	30.0–144.0

*Note*. BMI—body mass index, sTfR—soluble transferrin receptor, TIBC—total iron binding capacity

### Muscle strength (isometric conditions)

The maximal joint torque of the flexors and extensors of the elbow, shoulder, hip, knee and trunk were measured under isometric conditions. Special torque meters (Institute of Sport, Poland), type SMS1 (upper extremities) and SMS2 (lower extremities and trunk) [18], were used for measurements. Positions (angles, stabilization) in the measurement have been presented previously [18]. Joint torques of the right limb, left limb and trunk were measured separately (18 muscle groups), always in the order flexion-extension. Before any experimental testing, each individual completed a standardised warm-up. Each participant was required to develop a maximal contraction during assessment. Verbal encouragement was given to all participants to maintain their highest possible results. Joint torques were analyzed as maximal values obtained for each of the eighteen muscle groups and as the sum of joint torques of all muscle groups.

The total error in the measurement of the maximal joint torque did not exceed 4%. The maximal error of repeatability, expressed by the coefficient of variation, was 4.2%, while for the individual muscle groups it was 4.9% for hip flexors, 6.3% for shoulder extensors, and 1.8% and 2.1% for knee and hip extensors, respectively [19].

### Vertical jump performance

Vertical jumps on a Kistler force platform (amplifier Type 9281A, Switzerland) were used to measure the power output of lower extremities and calculate jump height. The amplifier was connected to a computer via an A/D converter. The MVJ v. 3.4 software package (“JBA” Zb. Staniak, Poland) was used for measurements. The maximal power (Pmax [W]), relative maximal power (*P*_max_·mass^-1^ [W·kg^-1^]) and maximal jump height (*h* [m]) were calculated from the registered ground reaction force of the platform [20].

Participants performed three counter-movement jumps (CMJs) and three spike jumps (SPJs) on the force platform. Athletes rested for five seconds between the CMJs and 1 min breaks between the SPJs. The participants were told to jump as high as possible in every trial and arm swing was allowed. The jump with the highest height achieved was chosen for statistical analysis.

The total error in the measurement of the maximal power output and jump height did not exceed 3.3% and 4.5%, respectively. The maximal error of repeatability, expressed by the coefficient of variation, for maximal power output was 3.4%, and 3.0% for jump height [19].

### Repeated sprint ability

The 5x6 repeated sprint cycle ergometry test on a Monark 874e with a friction load of 7.5% body mass was conducted to assess repeated sprint ability (RSA). Following a 5-minute warm up (about 70 W), each athlete was allowed a 5-minute recovery before performing the test. The 5x6 sprint cycle test comprised five 6-second maximal sprints commencing every 30 seconds. All sprints were performed from a stopped position and passive recovery was utilized between sprints. Five seconds before starting the next sprint, the athlete took a ready position and waited for the start. Verbal encouragement was given during the test to maintain their highest possible results [21].

An absolute (total kJ and W) and relative (J·kg^-1^ and W·kg^-1^) work and power score were calculated along with their respective decrement scores (% of decrement over repeated efforts). The equations for the decrement score calculation were as follows:

1. **Decrement of work**
  $$D_{W}=100-(\frac{W}{W^{\prime }∙5}*100)$$ (1)
  D_W_—decrement of work,
  *W*—total work,
  *W*′—highest 6 s work
2. **Decrement of power**
  $$D_{P}=100-(\frac{P_{min}}{P_{max}}*100)$$ (2)
  D_P_—decrement of power
  *P*_min_—lowest 6 s power,
  *P*_max_—highest 6 s power

### Anthropometric measurements

Body height was assessed using a SiberHegner anthropometer (Zurich, Switzerland) with an accuracy of 0.1 cm. Body mass (BM) and body composition were estimated by bioelectrical impedance (BIA) using a Tanita Body Composition Analyser MC-420 (Tokyo, Japan). During measurements, participants wore only underwear and remained barefoot.

### Sample collection and vitamin D and iron status

The blood samples were collected in the morning (8–9 am) from the antecubital vein in a seated position, after overnight fasting and a minimum of 12 hours after the last training session. In order to obtain the serum for testing, blood samples were centrifuged for 10 minutes at a speed of 3500 rpm. After centrifugation serum samples were stored frozen at -20°C prior to analysis.

The best measure of vitamin D status is the total concentration of serum 25-hydroxyvitamin D (25(OH)D) [22]. Total serum 25(OH)D concentration was analysed using commercially available ELISA kits (DiA Source, Louvain-La-Neuve, Belgium) according to the manufacturer’s protocol. All assays were performed in duplicate. The coefficient of variation of the intra-assays in this study was below 4% for 25(OH)D concentration. The 25OH Vitamin D Total ELISA (DiA Source, Louvain-La-Neuve, Belgium) has the Certificate of Proficiency issued by the Vitamin D External Quality Assessment Scheme (DEQAS) Advisory Panel [23].

Iron status was assessed using the following indices in serum: soluble transferrin receptor (sTfR) concentration using immunoenzymatic commercial kits (Ramco, Stafford, TX, USA); ferritin and iron concentration using the immunoturbidimetric method (Horiba ABX, Pentra 400, Monpellier cedex4, France); and total iron binding capacity (TIBC) using the colorimetric method (BioMaxima, Lublin, Poland). The coefficients of variation of the intra-assays in this study were below 4% for all iron status indices.

### Statistical analysis

All variables were inspected for univariate normality. Logarithmic transformations were performed on variables without normal distributions of the data. Pearson’s correlation coefficient was used to evaluate the associations between descriptive characteristics, 25(OH)D, iron status and exercise performance variables. Theory and an evaluation of the correlations between variables were used to identify potential confounding variables. Maturation, training experience, fat free mass (FFM), fat mass (FM) and height have been previously identified as correlates of exercise performance [24,25]. Partial correlation was used to evaluate the association between exercise performance and 25(OH)D and indices of iron status while adjusting for potential covariates.

25(OH)D concentrations were defined using the most commonly accepted definitions (25(OH)D < 20 ng·ml^-1^: deficiency, 20–30 ng·ml^-1^: insufficiency, above 30 ng·ml^-1^: sufficiency) [22]. Analysing 25(OH)D concentration based on deficiency did not significantly impact the results and was not presented. The level of significance was set at α = 0.05 and two-tailed tests were used.

## Results

The mean serum 25(OH)D concentration was 30.4 ng·ml^-1^ and ranged from 12.5 to 91.4 ng·ml^-1^. Eleven participants (22%) had vitamin D deficiency and 20 athletes (40%) had vitamin D insufficiency. Nineteen ice hockey players (38%) had 25(OH)D concentration above 30.0 ng·ml^-1^, including four participants had a serum vitamin D concentration >50 ng·ml^-1^.

A significant bivariate correlation was detected between 25(OH)D concentration and joint torque of the extensors of the right shoulder (*r* = -0.29, *p* = 0.04) and this association remained after adjusting for age, training experience, FFM, FM and height (*r* = -0.36, *p* = 0.02). Isometric strength for all other examined muscles groups (n = 17) and sum of joint torques were not associated with 25(OH)D concentration, before or after adjusting for potential covariates (*p*>0.05). Serum 25(OH)D concentration was correlated with mechanical power during the CMJ after adjusting for age, but not after adjusting for training experience, FFM, FM and height (Table 2). None of the measured exercise performance parameters differed significantly between sufficient, insufficient and deficient groups (data not shown).

**Table 2:** Pearson’s and partial correlations between exercise performance and (log of) 25(OH)D concentration (n = 50).

	Pearson’s	Partial Correlation	
		Model 1	Model 2
	*r*	*r*	*r*
Sum of muscle torque (N·m kg_bm_^-1^)	-0.14	-0.13	-0.21
CMJ height (cm)	0.21	0.27	0.22
SPJ height (cm)	0.23	0.26	0.18
CMJ power (W·kg_bm_^-1^)	0.25	0.29^a^	0.24
SPJ power (W·kg_bm_^-1^)	0.25	0.27	0.24
RSA total work (kJ)	-0.20	-0.17	-0.05
RSA relative total work (J·kg_bm_^-1^)	-0.10	-0.05	-0.05
RSA decrement of work (%)	0.07	0.06	0.06
RSA peak power (W)	-0.21	-0.18	-0.06
RSA relative peak power (W·kg_bm_^-1^)	-0.06	0.01	-0.05
RSA decrement of power (%)	-0.03	-0.02	0.02

*Note*. CMJ, countermovement jump; SPJ, spike jump; RSA, repeated sprint ability. Model 1: adjusted for age; Model 2: adjusted for age, training experience, fat mass, fat free mass and height.

^a^ indicates statistically significant at *p* < 0.05.

^b^ indicates trending at *p* < 0.10.

Serum 25(OH)D concentration was not associated with ferritin, iron, sTfR and TIBC (Table 3).

**Table 3:** Correlations between indices of iron status and (log of) 25(OH)D concentration (n = 50).

	*r*	*p*-value
Ferritin (ng·ml^-1^)	0.10	0.49
TIBC (μg·100ml^-1^)	-0.07	0.64
sTfR (μg·ml^-1^)	-0.01	0.95
Iron (μg·dl^-1^)	-0.06	0.66

*Note*. sTfR—soluble transferrin receptor, TIBC—total iron binding capacity.

Only iron concentration was correlated with sum of muscle torque for all muscle groups (Table 4). There was no statistically significant association between iron indices and power performance or RSA (data not shown).

**Table 4:** Pearson’s and partial correlations between exercise performance and Iron concentration (n = 50).

	Pearson’s	Partial Correlation	
		Model 1	Model 2
	*r*	*r*	*r*
Sum of muscle torque (N·m kg_bm_^-1^)	0.36^a^	0.36^a^	0.33^a^
CMJ height (cm)	-0.01	-0.02	-0.04
SPJ height (cm)	-0.04	-0.03	-0.03
CMJ power (W·kg_bm_^-1^)	-0.09	-0.09	-0.14
SPJ power (W·kg_bm_^-1^)	-0.01	-0.00	-0.03
RSA total work (kJ)	0.20	0.22	0.23
RSA relative total work (J·kg_bm_^-1^)	0.30^a^	0.35^a^	0.16
RSA decrement of work (%)	-0.07	-0.07	0.01
RSA peak power (W)	0.16	0.18	0.16
RSA relative peak power (W·kg_bm_^-1^)	0.19	0.24	0.08
RSA decrement of power (%)	0.03	0.03	0.02

*Note*. CMJ, countermovement jump; SPJ, spike jump; RSA, repeated sprint ability. Model 1: adjusted for age; Model 2: adjusted for age, training experience, fat mass, fat free mass and height.

^a^ indicates statistically significant at *p* < 0.05.

## Discussion

The main purpose of this study was to examine the association between serum 25(OH)D concentration, isometric strength, vertical jump performance and RSA in young ice hockey players. Contrary to our hypothesis, we did not detect a positive association between vitamin D concentration and studied exercise performance parameters (isometric strength, vertical jump performance and RSA) after adjusting for potential covariates. Serum 25(OH)D concentration was inversely related to isometric strength for one of 18 examined muscle groups. The direction and lack of a consitent statistically significant relationship seem to indicate 25(OH)D concentration is not associated with the strength of any muscle group examined in this study. No other associations between 25(OH)D and exercise performance remained statistically significant after adjusting for confounding. The secondary aim of this investigation was to evaluate the association between 25(OH)D concentration and indices of iron status. Iron status indices were not correlated with 25(OH)D concentration.

The association between vitamin D and exercise performance in athletes is not consistently observed in cross-sectional or experimental investigations. Lower levels of vitamin D were associated with lower grip strength in ice hockey players or lower jump height in football players [5,10]. Moreover, there was an increase of strength and power performance after supplementation of vitamin D in the group of ballet dancers [3] and judoka athletes [26]. In contrast to these studies, and according to our results, no correlation was found between vitamin D and muscle strength, muscle power or anaerobic capacity in football players [9] or swimmers [8].

In athletes, the relationship between 25(OH)D concentration and strength tends to be measured using hand-grip strength [8], isokinetic strength of the lower extremity [9,26] or isometric muscle strength of the dominant leg [3]. It was suggested that different skeletal muscle groups and fibre type predisposition might explain the discrepancy of the results between vitamin D status and lower- and upper-body maximal-intensity exercise [10]. Moreover, because of selective assessment of muscle groups, it is possible that vitamin D deficiency could influence muscle groups that were not examined in previous studies, resulting in a false negative observation [9]. In our study, muscle strength was measured in isometric conditions to determine maximal joint torque of the flexors and extensors of the elbow, shoulder, hip, knee and trunk. Despite that, we did not find a positive association between 25(OH)D concentration and isometric muscle strength for any muscle groups or for the sum of whole body muscle strength.

We also assessed exercise performance that is more closely linked to on-ice performance in ice hockey: vertical jump performance [25] and RSA [27]. Our results are consistent with Fitzgerald et al. [10], which failed to find a relationship between vitamin D concentration, vertical jump height, and power and fatigue index during the Wingate Test in ice hockey players. The lack of correlation between vitamin D concentration and exercise performance, especially sport-specific assessment, among young ice hockey players may be due to the benefits of training, which could mask the negative impact of vitamin D deficiency [9,10].

The secondary purpose of our study was to assess the relation between vitamin D and indices of iron status. According to Monlezun et al. [11], poor vitamin D status may increase cytokine production, which may result in increased hepcidin. It appears vitamin D supplementation may decrease hepcidin concentration, which may improve iron status [28]. It is well known that high concentration of hepcidin impairs absorption of iron in gastrointestinal tract [29]. Constantini et al. [16] showed, that iron depletion was higher in the athletes with lower serum 25(OH)D concentration. However in contrast to mentioned studies, a statistically significant association between iron status indices and 25(OH)D concentration was not observed in our athletes.

It is well known that iron deficiency may impair physical performance [14,15], that is heavily dependant on aerobic capacity [30]. In relation to anaerobic capacity, evidence suggests serum iron deficiency indices (ferritin, iron) are not associated with strength [31] or RSA [30]. In the present study, two key indices of iron status, i.e. serum ferritin and soluble transferrin receptor concentration were not associated with muscle strength, vertical jump performance or RSA [32]. The only iron indice correlation with muscle strength in studied ice hockey players was iron concentration, but due to of its high variability (large diurnal variation of around 30%) [33] the diagnostic utility of iron concentration in assessing iron status is rather low [34]. Because of it, the authors concluded that the relation between 25(OH)D concentration and exercise performance was neither facilitated nor supressed by the influence of iron status.

The lack of an association between 25(OH)D concentration, exercise performance and indcies of iron status in our athletes may be due to few possessing “optimal” or none with severely deficient vitamin D status. Fitzgerald et al. [10] suggested that the lack of association between vitamin D status and lower-body force and power production in ice hockey players could be related to a lack of athletes with deficient vitamin D status in their study. This is supported by the observation of other authors [35] that the impact of vitamin D on muscle function is more evident in athletes with significant vitamin D deficiency. In our study, 11 athletes had a deficiency (mean 17.3 ± 2.2 ng·ml^-1^) of vitamin D and 19 had a sufficient (mean 42.4 ± 17.9 ng·ml^-1^) concentration, so it seems that if there is a direct relationship between vitamin D and muscle function, it should be observed in this study. However, Heaney & Holick [36] indicated that 25(OH)D concentration should be as high as 48 to 90 ng·ml^-1^ for a perceptible physiological response of skeletal muscle as the optimal concentration of 25(OH)D may be tissue-specific [37]. Alternatively, severe vitamin D deficiency (<10 ng·ml^-1^) may be required to negatively impact muscle function. Both hypotheses may explain the lack of relationship between 25(OH)D concentration and exercise performance in our hockey players as few athletes had “optimal” (n = 4) and none with severely deficient vitamin D status.

Vitamin D insufficiency was observed in 62% of our athletes despite data collection taking place in close proximity to the hot and sunny summer. This finding is consistent with other investigations in outdoor and indoor athletes in Poland, Spain, Israel, England, USA and Tunisia [10,16,38,39,40,41]. It appears interventions aimed at increasing 25(OH)D concentration in athletes are needed to achieve and maintain sufficient status throughout the year. However, it should be noted, that 19 ice hockey players (38%) had 25(OH)D concentration above 30.0 ng·ml^-1^. Differences in dietary and supplemental vitamin D intake, sun exposure and genetics [42] are possible factors explaining the variability in 25(OH)D concentration. Unfortunately, we did not control for, or collect data on, these factors and cannot comment on the relative importance of each factor in achieving sufficient vitamin D status in our athletes. However, all athletes ate the same school canteen (only one main course, no vegan and vegetarian diet) [43] at least 4 weeks prior to data collection.

Our study has several limitations. This study was only powered (80%) to detect moderate correlations (0.38) between vitamin D status and exercise performance. Future investigations in athletes powered to detect weak associations or small effects are needed. We examined only male athletes, whom tend to have higher 25(OH)D concentrations than women [44], so it is desirable to perform a similar study in female athletes. Major strengths of our study in turn was that, all athletes were students from one ice hockey school and participated in similar type and volume of training; thus, it is unlikely that differences in training influenced our results. Furthermore they ate at the same school canteen and the study was conducted at a single time point in October. Therefore, confounding factors such as training, diet and seasonality were minimized.

In conclusion, we found vitamin D insufficiency to be highly prevalent in ice hockey players, but 25(OH)D concentration was not associated with exercise performance or indices of iron status. The failure to find significant relationships in this study may be due to few athletes possessing “optimal” and none had severely deficient vitamin D status. Future investigations should select cohorts and design interventions with this in mind to allow for the determination of the 25(OH)D concentration that corresponds to a change in exercise performance in athletes, if one exists. Due to the impact of vitamin D on many aspects of health, particularly bone health, it is important for athletes to remedy deficiency. Since deficiency appears common in athletes, especially those competing indoors, athletes should consider means of increasing vitamin D status through diet, supplements and sun exposure. Dose-response relationships in athletes participating in heavy exercise training need further attention.

## Supporting information

S1 Data PONE-D-17-45361 S1 Data.xlsx study results. (XLSX)
